# Toxic leadership, conscientious intelligence, and quality of nursing work life among registered nurses: A cross-sectional moderation study

**DOI:** 10.1016/j.ijnsa.2026.100662

**Published:** 2026-08-15

**Authors:** Ali D. Abousoliman, Mohamed Gamal Noaman Malek, Hagar Mahmoud Hamed

**Affiliations:** a College of Nursing, Prince Sattam bin Abdulaziz University, Al-Kharj, Saudi Arabia; b Nursing Administration, Faculty of Nursing, KafrElsheikh University, Egypt

**Keywords:** Conscientious intelligence, Toxic leadership, Quality of work life, Nurses, Leadership

## Abstract

**Background:** Quality of nursing work life is an important indicator of nurses’ professional well-being. Toxic leadership has been associated with poorer nursing outcomes; however, limited evidence exists on how personal ethical resources, such as conscientious intelligence, may shape the relationship between toxic leadership and quality of nursing work life among registered nurses. **Aim:** We aimed to examine the relationship between toxic leadership and quality of nursing work life among registered nurses and to determine whether conscientious intelligence moderated this relationship. **Design:** A cross-sectional analytical design was used. **Methods:** A convenience sample of 274 registered nurses in Egypt completed self-administered questionnaires assessing the study variables. Schmidt’s Toxic Leadership Scale, the Conscientious Intelligence Scale, and the Quality of Nursing Work Life Scale were used. Data were analyzed using descriptive statistics, independent samples *t*-test, one-way analysis of variance, Pearson’s correlation, and Hayes’ PROCESS Model 1 for moderation analysis. **Results:** Toxic leadership was negatively correlated with quality of nursing work life (*r* = −.605, *p* < .001) and conscientious intelligence (*r* = −.334, *p* < .001)**.** Conscientious intelligence was positively correlated with quality of nursing work life (*r* = .318, *p* < .001). The moderation model was statistically significant, *F*(3, 270) = 59.026, *p* < .001, and explained 39.6% of the variance in quality of nursing work life. The interaction was significant (*B* = −0.0065, standard error = 0.0026**,***t* = −2.538, *p* = .012) and explained an additional 1.4% of the variance. The negative conditional association between toxic leadership and quality of nursing work life became stronger as conscientious intelligence increased. **Conclusion:** Greater perceived toxic leadership was associated with poorer quality of nursing work life. Although conscientious intelligence was positively associated with quality of nursing work life at the bivariate level, it did not demonstrate the hypothesized protective moderation. The stronger negative association at higher levels of conscientious intelligence may reflect greater sensitivity to perceived violations of professional and moral values, but this explanation requires further investigation. **Implications for Nursing Management:** Healthcare organizations should consider prioritizing leadership evaluation, accountability systems, safe reporting mechanisms, and efforts to strengthen ethical climates. Initiatives to strengthen nurses’ conscientious and ethical capacities could be accompanied by organizational efforts to create ethically supportive work environments.

**What is already known about the topic**

• Toxic leadership is associated with poorer nurse outcomes, including reduced job satisfaction, lower engagement, emotional exhaustion, and turnover intention.
• Quality of nursing work life is an important indicator of nurses’ professional well-being, retention, and perceived work environment.
• Conscientious intelligence is related to nurses’ ethical awareness, moral sensitivity, professional responsibility, and humanistic care.

Alt-text: Unlabelled box dummy alt text

**What this paper adds**

• There was a strong negative association between perceived toxic leadership and quality of nursing work life among a sample of registered nurses.
• Conscientious intelligence statistically moderated the relationship between perceived toxic leadership and quality of nursing work life.
• Contrary to the hypothesized protective pattern, the negative association between perceived toxic leadership and quality of nursing work life was stronger among participants with higher conscientious intelligence.

Alt-text: Unlabelled box dummy alt text

## 1 Introduction

Registered nurses (RNs) form the largest professional group in healthcare systems worldwide and play a central role in patient care delivery (World Health Organization, 2020). Quality of nursing work life is a multidimensional construct reflecting the extent to which nurses’ work and work environments meet their personal and professional needs (Brooks and Anderson, 2005). Higher quality of nursing work life has been associated with a lower intention to leave the profession and a lower likelihood of subsequently leaving it (Lee et al., 2017). Given persistent nursing workforce shortages, identifying factors associated with quality of nursing work life is important for workforce sustainability (World Health Organization, 2020; Tamata and Mohammadnezhad, 2023).

Contemporary healthcare environments are characterized by persistent workload pressures, staffing shortages, escalating patient acuity, and emotional demands that may adversely affect nurses’ work experiences (World Health Organization, 2020; Tamata and Mohammadnezhad, 2023). Leadership practices of nurse managers and supervisors are associated with the psychological and relational conditions in which nurses practice, including their motivation, autonomy, psychological safety, and professional commitment (Bakker and Demerouti, 2017; Conroy et al., 2023). Transformational leadership has been associated with staff nurse retention (Conroy et al., 2023). In contrast, toxic leadership has been associated with greater conflict, lower organizational commitment, and stronger turnover intentions (Alsadaan and Alqahtani, 2024; Ofei et al., 2023).

Researchers studying nurses have reported associations between perceived toxic leadership and emotional exhaustion, lower job satisfaction, psychological distress, and turnover intention (Ahmed et al., 2024; Labrague, 2024; Mrayyan, 2025; Ofei et al., 2023). However, the possible role of conscientious intelligence in this relationship remains unclear. Conscientious intelligence is a multidimensional construct concerning the ethical, moral, reflective, and socially sensitive-aspects of conscience (Aktı et al., 2017). We were unable to find evidence associating conscientious intelligence with the quality of nursing work life or as a moderator for perceived toxic leadership among RNs. Therefore, we aimed to examine the associations among perceived toxic leadership, conscientious intelligence, and quality of nursing work life and to test whether the association between perceived toxic leadership and quality of nursing work life varied according to conscientious intelligence.

The study objectives were to:

• Assess the levels of perceived toxic leadership, quality of nursing work life, and conscientious intelligence;
• Examine the associations among perceived toxic leadership, quality of nursing work life, and conscientious intelligence; and
• Test whether conscientious intelligence statistically-moderated the association between perceived toxic leadership and quality of nursing work life among registered nurses.

## 2 Literature review

Quality of nursing work life extends beyond conventional job satisfaction by considering whether nurses’ work and working environments meet their personal and professional needs while supporting organizational goals. Brooks and Anderson (2005) described four interrelated domains: work life-home life, which concerns the interface between professional and personal responsibilities; work design, which concerns the nature and organization of nursing work; work context, which encompasses the practice environment, resources, and professional relationships; and work world, which reflects broader societal and policy influences on nursing. Researchers have reported associations between lower quality of nursing work life and stronger intention to leave, while prospective evidence has linked it to subsequent departure from the nursing profession (Faraji et al., 2017; Lee et al., 2017). Because the work-context domain includes supervisory relationships and characteristics of the practice environment, quality of nursing work life is a relevant outcome for examining nurses’ perceptions of leadership.

Schmidt (2008) operationalized toxic leadership through five behavioral dimensions: abusive supervision, authoritarian leadership, narcissism, self-promotion, and unpredictability. Researchers studying nurses have reported associations between perceived toxic leadership and stronger turnover intentions, lower job satisfaction and engagement, greater psychological distress, workplace conflict, and lower organizational commitment (Ofei et al., 2023; Alsadaan and Alqahtani, 2024; Labrague, 2024; Mrayyan, 2025). Several of these studies used cross-sectional designs; therefore, their findings demonstrate associations but cannot establish temporal order or causality. Nevertheless, the consistency of the reported associations provides a rationale for examining toxic leadership in relation to the broader construct of quality of nursing work life.

Researchers in Egypt reported a negative association between toxic leadership and nurses’ quality of work life and found that this association varied according to nurses’ agility (resilience and adaptability) (Tawfik et al., 2025). However, those researchers used the general Work-Related Quality of Life Scale rather than Brooks’ nursing-specific Quality of Nursing Work Life Survey and did not examine conscientious intelligence. Consequently, evidence concerning the association between toxic leadership and nursing-specific quality of work life and whether this association varies according to conscientious intelligence remains limited.

Aktı et al. (2017) conceptualized conscientious intelligence as a multidimensional construct encompassing ethical values, moral sensitivity, responsibility toward the creator, compassion, conscious awareness, social sensitivity, and wisdom. This construct was originally validated among 1546 adults with varied demographic characteristics living in Kütahya, Türkiye; it was not developed specifically for nurses (Arslanoğlu et al., 2024). Research applying the construct in nursing has primarily involved nursing students. Dur et al. (2022) examined its relationship with cultural sensitivity, while Özcan and Yanmış (2022) compared conscientious-intelligence levels according to year of nursing education and participant characteristics. Neither study examined RNs’ quality of nursing work life or their perceptions of toxic leadership.

Within Job Demands-Resources theory, job demands refer to aspects of work that require sustained effort and are associated with psychological or physiological costs, whereas resources support goal achievement, reduce the costs associated with demands, or facilitate development (Bakker and Demerouti, 2017). In the present study, we conceptualized perceived toxic leadership as an adverse social job demand because it represents an unfavorable interpersonal and organizational condition. We conceptualized conscientious intelligence as a personal resource because its ethical, reflective, and socially sensitive dimensions may be relevant to how nurses interpret and respond to workplace experiences. This classification represents the theoretical proposition tested in this study, rather than an empirically established property of conscientious intelligence.

Statistical moderation occurs when the magnitude or direction of the association between two variables differs according to the level of a third variable (Baron and Kenny, 1986). Evidence involving resources analogous to conscientious intelligence has been mixed. In contrast, Koç et al. (2022) found no statistical evidence that psychological well-being moderated the association between toxic leadership and nurses’ vengeful behavior. Because both studies were cross-sectional and examined constructs other than conscientious intelligence, they provide only indirect support for a possible moderating association and do not establish a protective mechanism.

Competing theoretical expectations are also plausible. Conscientious intelligence may provide nurses with an ethical and reflective framework for interpreting adverse workplace experiences. Conversely, greater moral sensitivity may make perceived violations of professional values more salient, particularly in an unsupportive ethical climate. This latter possibility is consistent with evidence of a moderate negative association between ethical climate and moral distress among nurses (Xue et al., 2025). However, this evidence does not directly demonstrate how conscientious intelligence operates in toxic leadership situations. Thus, conscientious intelligence should not be assumed to function as a universally-protective resource, and its possible moderating association requires empirical testing.

Hypotheses Accordingly, we hypothesized that perceived toxic leadership was negatively associated with quality of nursing work life (H1); conscientious intelligence was positively associated with quality of nursing work life (H2); and conscientious intelligence statistically-moderated the association between perceived toxic leadership and quality of nursing work life, such that the negative association was weaker among RNs with higher conscientious intelligence (H3).

## 3 Methods

### 3.1 Research design

A quantitative cross-sectional analytical design was used to examine associations among perceived toxic leadership, conscientious intelligence, and quality of nursing work life among RNs. This design was appropriate for testing whether conscientious intelligence statistically-moderated the association between toxic leadership and quality of nursing work life using moderation analysis.

### 3.2 Setting

The study was conducted at Kafr Elsheikh University Hospital in Egypt. University Hospital is a multidisciplinary teaching hospital affiliated with Kafr Elsheikh University and provides educational and clinical services across a range of specialties, with a total capacity of 434 beds. It includes intensive care beds, operating rooms, radiology services, laboratories, blood bank services, and dialysis units. RNs working in different clinical departments and units within the hospital were eligible to participate in the study.

### 3.3 Participants and sampling

A convenience sample of RNs working at the study hospital was recruited. Eligible participants were licensed RNs who were actively employed during data collection and had at least 1 year of professional experience. Nurses who were on leave, completing an internship, or unavailable during data collection were excluded.

The required sample size was determined a priori using G*Power software version 3.1.9.4 for multiple linear regression analysis. The calculation was based on a small effect size of 0.02, a significance level of 0.05, and statistical power of 0.80, indicating a minimum required sample of 271 participants. To compensate for incomplete or unusable responses, additional nurses were invited to participate. A total of 274 RNs completed the questionnaires and were included in the final analysis.

### 3.4 Data collection methods

Data were collected using a self-administered questionnaire composed of four sections: demographic characteristics, toxic leadership, conscientious intelligence, and quality of nursing work life.

### 3.5 Demographic characteristics

The first section included questions related to participants’ demographic and professional characteristics, including age, sex, marital status, educational qualification, department/unit, and years of professional nursing experience.

### 3.6 Toxic leadership scale

Toxic leadership was assessed using the 30-item Toxic Leadership Scale developed by Schmidt (2008). The scale consists of five subdimensions: abusive supervision, authoritarian leadership, narcissism, self-promotion, and unpredictability. Responses are rated on a 6-point Likert scale ranging from 1 = strongly disagree to 6 = strongly agree. Higher scores indicate higher perceived toxic leadership. In the present study, the scale demonstrated excellent internal consistency, with a Cronbach’s alpha coefficient of .91.

### 3.7 Conscientious intelligence scale

Conscientious intelligence was measured using the 32-item Conscientious Intelligence Scale developed by Aktı et al. (2017). The scale includes four subdimensions: Spiritual Direction, Emotional/Social Conscientiousness, Psychological Conscientiousness, and Moral/Universal Conscientiousness. Items were rated on a five-point Likert scale ranging from 1 = strongly disagree to 5 = strongly agree. Items 1–5 were reverse-scored, producing a possible total score ranging from 32 to 160, with higher scores indicating greater conscientious intelligence. In the original validation study, Cronbach’s alpha was .85 for the total scale, and the subdimension coefficients ranged from .63 to .85. In the present study, Cronbach’s alpha for the total scale was .88.

### 3.8 Quality of nursing work life scale

Quality of nursing work life was measured using the Quality of Nursing Work Life Scale developed by Brooks and Anderson (2005). The scale consists of 42 items distributed across four domains: Work Life/Home Life, Work Design, Work Context, and Work World. Items are rated on a 6-point Likert scale ranging from 1 = strongly disagree to 6 = strongly agree. Higher scores indicate a more favorable perception of quality of nursing work life. Previous studies have reported strong internal consistency for the subscales, with Cronbach’s alpha coefficients ranging from .88 to .92. In the current study, the scale was used to assess nurses’ perceptions of their work environment, work-life balance, professional context, and broader work-related conditions.

### 3.9 Data collection procedure

Ethical approval for the study was obtained from the Research Ethics Committee, with approval number KFSIRB400–67, on May 27, 2025. Administrative approval was subsequently obtained from Kafr Elsheikh University Hospital. Data were collected between June 15 and August 2, 2025.

Data were collected by members of the research team using a standardized data collection procedure. Before data collection, the researchers agreed on a uniform explanation of the study purpose, eligibility criteria, informed consent process, questionnaire distribution, and questionnaire return procedure to ensure consistency across participants and units. Eligible nurses were approached in their respective units at suitable times that did not interfere with patient care. The researchers explained the purpose of the study, participation requirements, voluntary nature of participation, confidentiality of responses, and the right to withdraw at any time without penalty. Nurses who agreed to participate and provided written informed consent received the questionnaire packet in a sealed envelope. Participants completed the questionnaire independently, and completion required approximately 12–15 min. Completed questionnaires were returned to the research team in sealed envelopes. Sealed envelopes were used to protect participants’ privacy, reduce social desirability bias, and reassure nurses that their responses could not be viewed by managers, colleagues, or members of the research team during collection.

### 3.10 Ethical considerations

The study was conducted in accordance with the ethical principles of the Declaration of Helsinki. Approval was obtained from the relevant Research Ethics Committee and hospital administration before data collection. Participation was voluntary, and all participants were informed about the study purpose, procedures, expected completion time, confidentiality of responses, and their right to withdraw at any time without penalty. Written informed consent was obtained from all participants before completing the questionnaire. No identifying information was collected, and all data were coded and stored securely to maintain confidentiality and anonymity.

### 3.11 Statistical analysis

Data were analyzed using IBM SPSS Statistics version 27.0. Descriptive statistics, including frequencies, percentages, means, and standard deviations, were used to summarize participants’ demographic characteristics and the main study variables. The internal consistency reliability of the study instruments was assessed using Cronbach’s alpha coefficient, with values of .70 or above considered acceptable.

Independent samples *t*-tests and one-way analysis of variance (ANOVA) were used to examine differences in toxic leadership, conscientious intelligence, and quality of nursing work life according to demographic and professional characteristics. Pearson’s correlation coefficient was used to examine the bivariate relationships among toxic leadership, conscientious intelligence, and quality of nursing work life.

To test the moderating role of conscientious intelligence, moderation analysis was conducted using Hayes’ PROCESS macro for SPSS, Model 1. Toxic leadership was entered as the independent variable, quality of nursing work life as the dependent variable, and conscientious intelligence as the moderating variable. The interaction term between toxic leadership and conscientious intelligence was examined to determine whether conscientious intelligence significantly altered the strength or direction of the relationship between toxic leadership and quality of nursing work life. Continuous variables were mean-centered before generating the interaction term. A significant interaction effect was interpreted as evidence that conscientious intelligence moderates the relationship between toxic leadership and quality of nursing work life. Statistical significance was set at *p* < .05.

## 4 Results

Because this study used a cross-sectional design, the findings should be interpreted as associations rather than causal effects. No causal inference is intended. Differences in toxic leadership, quality of nursing work life, and conscientious intelligence were examined according to participants’ demographic characteristics. Independent samples *t*-test was used for sex, whereas one-way ANOVA was used for age, marital status, educational level, and years of experience. Findings related to age and educational-level differences should be interpreted cautiously because some subgroups were very small, particularly nurses aged 51 years or older and nurses holding a PhD degree.

As shown in Table 1, no statistically significant sex differences were found in toxic leadership, quality of nursing work life, or conscientious intelligence. Similarly, marital status and years of experience were not significantly associated with any of the three study variables.

**Table 1:** Differences in toxic leadership, quality of nursing work life, and conscientious intelligence by participants’ demographic characteristics (*N*= 274).

Demographic characteristics	Group	N	Toxic leadership			Quality of nursing work life			Conscientious intelligence		
			Mean	S. D	Test of sig.	Mean	S. D	Test of sig.	Mean	S. D	Test of sig.
**Age**	<30	170	58.41	16.96	F = 0.975 *p* = .405	161.53	10.63	F = 2.681 *p* = .047	104.88	8.64	F = 0.953 *p* = .416
	From 31–40	90	61.70	17.45		160.60	10.25		105.81	8.25	
	From 41–50	11	55.27	12.26		152.55	12.27		106.00	9.72	
	≥51	3	57.33	15.04		165.33	12.86		112.33	2.89	

**Sex**	Male	70	57.34	16.00	*t* = −1.151 *p* = .251	160.11	10.63	*t* = −0.717 *p* = .474	105.10	9.29	*t* = −0.239 *p* = .812
	Female	204	60.04	17.26		161.18	10.72		105.38	8.27	
**Marital Status**	Single	84	60.26	18.51	F = 0.452 *p* = .716	160.38	10.45	F = 0.857 p = .464	105.45	8.00	F = 1.919 p = .127
	Married	155	58.61	16.79		161.66	10.83		105.82	8.30	
	Divorced	19	62.63	14.48		158.00	9.43		104.53	11.51	
	Widowed	16	57.94	12.91		159.75	12.01		100.56	8.61	

**Educational Level**	Diploma	90	59.16	15.75	F = 0.586 *p* = .624	162.14	10.03	F = 5.155 *p* = .002	106.51	7.83	F = 1.843 *p* = .140
	Bachelor	163	59.89	18.11		161.27	10.38		104.74	8.48	
	Master	17	57.65	13.08		154.00	13.63		103.06	11.73	
	PhD	4	49.25	3.50		147.50	6.25		111.00	6.78	
**Experience Years**	1–5	134	59.73	17.65	F = 0.287 *p* = .835	161.85	10.08	F = 1.136 *p* = .335	105.03	8.82	F = 0.389 *p* = .761
	6–10	84	59.57	17.68		159.99	11.90		105.02	8.48	
	11–15	43	59.00	14.36		160.93	9.08		106.44	7.53	
	≥16	13	55.23	13.63		157.00	13.10		106.31	9.38	

**Note**. S.D = standard deviation. Independent samples *t*-test was used for gender, whereas one-way ANOVA was used for age, marital status, educational level, and years of experience.

Regarding age, no significant differences were found in toxic leadership or conscientious intelligence. However, quality of nursing work life differed significantly according to age group. Nurses aged ≥51 years had the highest mean quality of nursing work life score, whereas nurses aged 41–50 years had the lowest mean score.

Educational level was not significantly associated with toxic leadership or conscientious intelligence. However, quality of nursing work life differed significantly according to educational level. Nurses with diploma/technical education reported the highest mean quality of nursing work life score, whereas nurses with PhD degrees reported the lowest mean score.

Overall, demographic characteristics were not significantly associated with toxic leadership or conscientious intelligence. Significant differences were found only in quality of nursing work life according to age and educational level.

As shown in Table 2, the overall level of perceived toxic leadership was low, with all five dimensions falling within the low range. Unpredictability was the most prominent toxic leadership dimension, whereas authoritarian leadership was the least prominent. The overall level of conscientious intelligence was moderate. Among its four dimensions, psychological conscientiousness was high; spiritual direction and emotional/social conscientiousness were moderate; and moral/universal conscientiousness was low. The overall level of quality of nursing work life was moderate, with all four domains falling within the moderate range. Work life/home life had the highest mean item score, whereas work world had the lowest.

**Table 2:** Descriptive statistics of study variables among nurses.

Variable / Dimension	Minimum	Maximum	Mean	Std. Deviation
Toxic abusive leadership	7.00	35.00	14.66	5.90
Toxic authoritarian leadership	6.00	26.00	10.58	4.35
Toxic narcissism	5.00	21.00	8.91	3.65
Toxic self-promotion	5.00	25.00	9.38	3.70
Toxic unpredictability	7.00	35.00	15.82	6.11
**Overall toxic leadership**	**33.00**	**139.00**	**59.35**	**16.96**
Spiritual conscientious intelligence	17.00	42.00	31.44	4.40
Emotional-social conscientious intelligence	12.00	40.00	29.12	4.07
Psychological conscientious intelligence	17.00	35.00	26.14	3.69
Moral conscientious intelligence	8.00	27.00	18.62	3.47
**Overall Conscientious intelligence**	**71.00**	**126.00**	**105.31**	**8.52**
Work-home life	24.00	52.00	37.39	5.69
Work design	21.00	66.00	41.51	6.61
Work context	28.00	61.00	44.59	5.63
Work world	22.00	51.00	37.41	5.34
**Overall quality of nursing work life**	**129.00**	**187.00**	**160.91**	**10.69**

As shown in Table 3, Pearson’s correlation analysis was conducted to examine the relationships among toxic leadership, conscientious intelligence, and quality of nursing work life. Toxic leadership was significantly and negatively correlated with conscientious intelligence and quality of nursing work life. This indicates that higher perceived toxic leadership was associated with lower conscientious intelligence and poorer quality of nursing work life.

**Table 3:** Correlations among toxic leadership, conscientious intelligence, and quality of nursing work life.

Variables	Toxic leadership	Conscientious intelligence
**Toxic leadership**	1	

**Conscientious intelligence**	−.334**	1
**Quality of nursing work life**	−.605**	.318**

**Note**. **p < .001.

Conscientious intelligence was significantly and positively correlated with quality of nursing work life, suggesting that nurses with higher conscientious intelligence reported better quality of nursing work life. Accordingly, the first hypothesis, which proposed that toxic leadership was negatively associated with quality of nursing work life, was supported. The second hypothesis, which proposed that conscientious intelligence was positively associated with quality of nursing work life, was also supported.

As shown in Table 4, Panel A, the moderation model was statistically significant and explained 39.6% of the variance in quality of nursing work life. The interaction between toxic leadership and conscientious intelligence was statistically significant and explained an additional 1.44% of the variance. Thus, statistically significant moderation was observed; however, H3 was not supported because the direction of the interaction was opposite to the hypothesized protective pattern.

**Table 4:** Moderation model and conditional associations for quality of nursing work life.

Panel A. Moderation model						
Predictor	Unstandardized coefficient	Standard error	*t*	*p*	95% confidence interval, lower	95% confidence interval, upper
Constant	126.815	16.701	7.593	< .001	93.934	159.696
Toxic leadership	0.310	0.264	1.175	.241	−0.209	0.829
Conscientious intelligence	0.534	0.159	3.359	.001	0.221	0.847
Toxic leadership × conscientious intelligence	−0.0065	0.0026	−2.538	.012	−0.0116	−0.0015
**Panel B. Conditional associations**						
**Level of conscientious intelligence**	**Conscientious intelligence value**	**Conditional coefficient of toxic leadership**	**Standard error**	***t***	***p***	**95% confidence interval**
Low	96.786	−0.322	0.034	−9.469	< .001	(−0.389, −0.255)
Mean	105.310	−0.378	0.033	−11.462	< .001	(−0.443, −0.313)
High	113.834	−0.434	0.045	−9.749	< .001	(−0.521, −0.346)

**Model summary.***R* = .629, *R*^2^ = .396, mean squared error = 69.750, *F*(3, 270) = 59.026, *p* < .001. Interaction. Change in *R*^2^ = .0144, *F*(1, 270) = 6.441, *p* = .012.

**Note**. Low and high conscientious intelligence values represent 1 standard deviation below and above the mean, respectively. All coefficients are unstandardized; confidence intervals are 95%.

As shown in Table 4, Panel B, toxic leadership was negatively associated with quality of nursing work life at low, mean, and high levels of conscientious intelligence. The magnitude of this negative association increased as conscientious intelligence increased. Therefore, conscientious intelligence did not attenuate the negative association; instead, a stronger negative association was observed at higher levels of conscientious intelligence.

Because the model included an interaction term, the lower-order coefficient for toxic leadership was conditional on the reference value of conscientious intelligence and was not interpreted as an overall association. Interpretation therefore focused on the interaction term and the conditional associations.

**Fig. 1:**
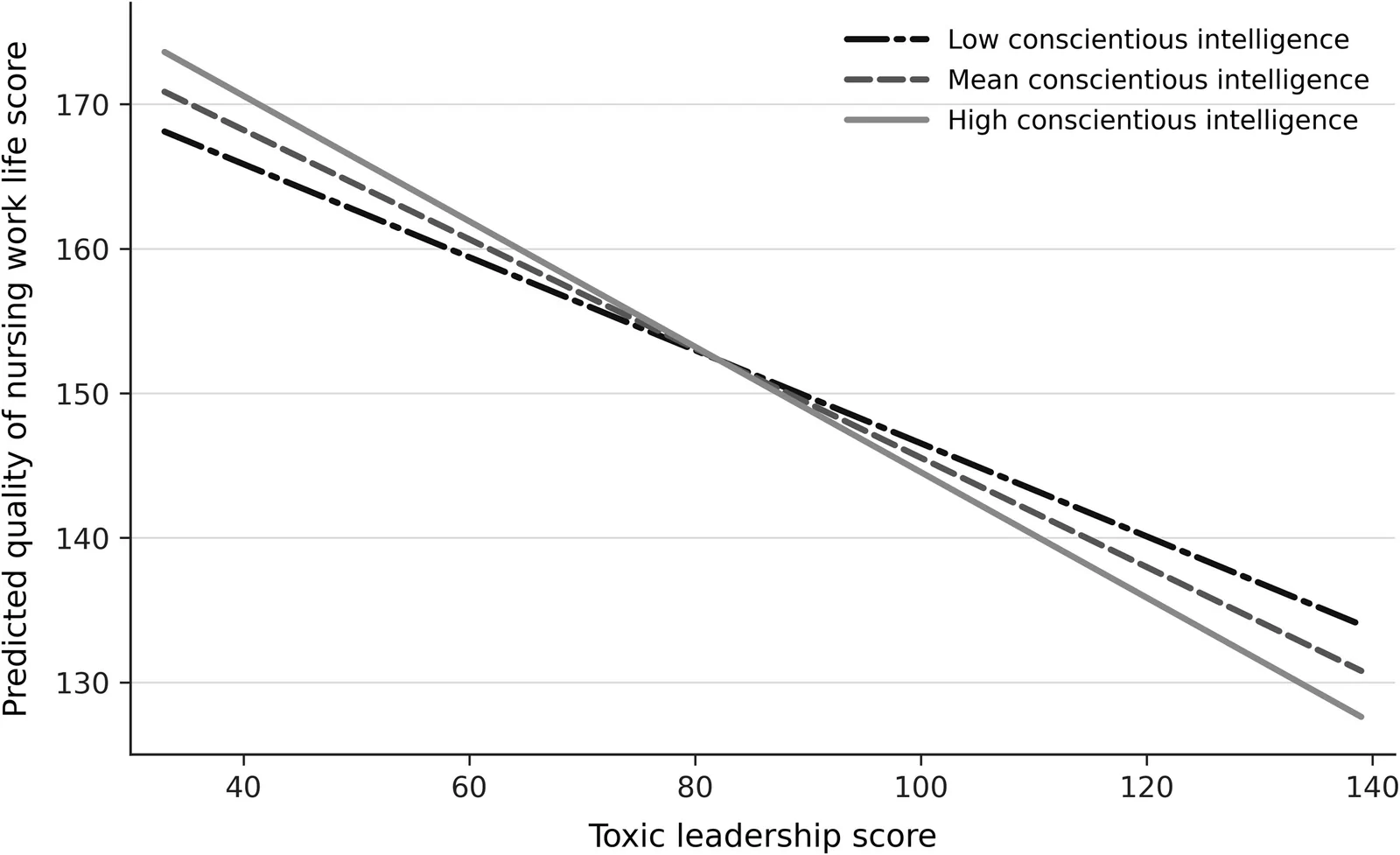
Conditional association between toxic leadership and quality of nursing work life at low, mean, and high levels of conscientious intelligence. Note. Low, mean, and high levels of conscientious intelligence correspond to one standard deviation below the mean, the mean, and one standard deviation above the mean, respectively.

As shown in Fig. 1, toxic leadership was negatively associated with quality of nursing work life at low, mean, and high levels of conscientious intelligence. The slope became progressively more negative as conscientious intelligence increased, indicating that the negative association was strongest among participants with higher conscientious intelligence.

## 5 Discussion

We examined the associations of toxic leadership and conscientious intelligence with quality of nursing work life among the participants and whether the association between toxic leadership and quality of nursing work life differed across levels of conscientious intelligence. Toxic leadership was negatively associated with quality of nursing work life. At the bivariate level, conscientious intelligence was positively associated with quality of nursing work life. The statistically significant interaction indicated that conscientious intelligence moderated the association between toxic leadership and quality of nursing work life; however, the direction of the moderation was opposite to the hypothesized protective pattern.

The first hypothesis was supported at the bivariate level because perceived toxic leadership was negatively associated with quality of nursing work life. This association indicated that participants reporting greater toxic leadership also reported poorer quality of nursing work life. This result is consistent with Tawfik et al. (2025), who reported a significant negative relationship between toxic leadership and quality of work life among nurses, and with Ofei et al. (2023), who found that toxic leadership among nurse managers was associated with reduced job satisfaction and higher turnover intention. Similarly, Mrayyan (2025) found that toxic leadership was negatively related to workplace satisfaction and job engagement among RNs. We have extended this evidence by demonstrating that toxic leadership was associated not only with specific outcomes, such as job satisfaction and turnover intention, but also with the broader construct of quality of nursing work life.

This relationship may be explained by the multidimensional nature of quality of nursing work life. Brooks and Anderson (2005) conceptualized quality of nursing work life as including work life/home life, work design, work context, and work world, while Brooks et al. (2007) emphasized that quality of nursing work life reflects the broader conditions shaping nurses’ professional experience. Perceptions of toxic leadership behaviors, including abusive supervision, authoritarianism, narcissism, self-promotion, and unpredictability, may coincide with lower autonomy, weaker trust, greater stress, and less stable working conditions (Schmidt, 2008). Ahmed et al. (2024) also reported associations among toxic leadership, emotional exhaustion, and adverse workplace behavior. We have provided context for the observed association, although the present cross-sectional design cannot establish the mechanisms through which toxic leadership and quality of nursing work life may be related.

The second hypothesis was supported at the bivariate level because conscientious intelligence was positively associated with quality of nursing work life. Conscientious intelligence encompasses ethical awareness, spiritual direction, emotional and social conscientiousness, psychological conscientiousness, and moral and universal conscientiousness. These attributes may be associated with a stronger sense of professional meaning, commitment, and positive workplace relationships. Researchers have linked conscientious intelligence with cultural sensitivity, empathy, and spiritual care (Aktı et al., 2017; Dur et al., 2022; Özcan and Yanmış, 2022). Aghakhani et al. (2025) also discussed the relevance of moral sensitivity to ethical decision-making and professional responsibility among nurses. Nevertheless, the positive bivariate association should not be interpreted as evidence that conscientious intelligence necessarily protected participants from the negative association between toxic leadership and quality of nursing work life.

Toxic leadership and conscientious intelligence were also negatively correlated. Participants who reported greater toxic leadership reported lower conscientious intelligence. Because both variables were self-reported and measured concurrently, this relationship cannot establish whether perceived toxic leadership influenced conscientious intelligence, whether conscientious intelligence affected the recognition or reporting of toxic behavior, or whether both were associated with other unmeasured factors. Abou Hashish and Razek (2025) reported associations among an unsupportive ethical climate, moral distress, and poorer well-being. This provides a possible contextual interpretation of the inverse correlation, but ethical climate and moral distress were not measured in the present study.

We consider the most important finding to be the statistically-significant interaction between toxic leadership and conscientious intelligence. We hypothesized that conscientious intelligence would weaken the negative association between toxic leadership and quality of nursing work life. Although the interaction was statistically significant, its direction was contrary to this proposed protective pattern. The negative association between toxic leadership and quality of nursing work life was significant at low, mean, and high levels of conscientious intelligence and became progressively stronger as conscientious intelligence increased. Therefore, the third hypothesis was not supported in the hypothesized direction.

The plotted estimates in Fig. 1 further showed that higher conscientious intelligence corresponded to higher predicted quality of nursing work life at lower toxic leadership scores but to lower predicted quality of nursing work life at higher toxic leadership scores. This crossover should not be interpreted as indicating that conscientious intelligence was inherently detrimental. Instead, it indicates that its association with quality of nursing work life varied according to the level of perceived toxic leadership. The observed pattern was, therefore, more consistent with amplification of the negative association than with protective moderation.

This result differs from Tawfik et al. (2025), who reported a moderating role for nurses’ agility in the negative relationship between toxic leadership and quality of work life. The difference may relate to the distinct characteristics represented by the two moderators. Agility reflects adaptability, flexibility, and adjustment to workplace demands, whereas conscientious intelligence emphasizes moral awareness and ethical sensitivity. Participants with higher conscientious intelligence may, therefore, be more attentive to discrepancies between leaders’ behavior and professional values. This explanation remains provisional and requires direct examination in future research.

A related interpretation concerns ethical value incongruence. Participants with strong moral awareness may experience greater discomfort when they perceive that leaders’ behavior conflicts with professional and ethical values. Yang et al. (2023) reported relationships among moral distress, resilience, and moral courage among nurses, while Abou Hashish and Razek (2025) and Xue et al. (2025) highlighted the relevance of ethical climate to nurses’ moral distress and well-being. This literature provides a basis for proposing that conscientious intelligence may be advantageous in supportive environments but may coincide with greater distress when leadership behavior conflicts with professional values. However, because ethical climate, value incongruence, and moral distress were not measured, this interpretation should be tested rather than treated as an established explanation.

Although the interaction was statistically significant, its incremental contribution to the explained variance was modest. The interaction should therefore be viewed as a meaningful qualification of the overall association rather than as its dominant explanation. The results also indicate that conscientious intelligence should not be assumed to function as a sufficient personal buffer against perceived toxic leadership. Organizational conditions should be considered alongside individual moral resources when interpreting quality of nursing work life.

There were no statistically significant differences in toxic leadership, conscientious intelligence, or quality of nursing work life according to sex, marital status, or years of experience. These non-significant results do not demonstrate that the groups were equivalent because limited subgroup sizes may have reduced the ability to detect differences. Quality of nursing work life differed according to age and educational level. Participants aged 51 years or older reported the highest quality of nursing work life, whereas those aged 41–50 years reported the lowest. Participants with diploma or technical qualifications reported the highest scores, whereas those with doctoral qualifications reported the lowest. These comparisons should be interpreted cautiously because some age and educational subgroups were small. Although differences in career stage, professional expectations, autonomy, and role utilization may provide possible explanations (Faraji et al., 2017), the present findings cannot establish why these group differences occurred.

### 5.1 Implications for nursing management

Healthcare organizations should consider establishing systems for identifying and addressing toxic leadership behaviors through leadership evaluation, confidential reporting mechanisms, coaching, mentoring, and accountability policies (Mrayyan, 2025; Ofei et al., 2023). They could also consider establishing clear behavioral standards for nurse managers, incorporating indicators of abusive and authoritarian behavior into performance appraisal systems, and ensuring that complaints are investigated fairly. Hospital leaders should also consider supporting psychological safety, access to ethical consultation, and protection from retaliation for participants who report harmful leadership behavior.

Given that the negative association between toxic leadership and quality of nursing work life was stronger at higher levels of conscientious intelligence, organizations could also consider providing ethically sensitive staff with opportunities for professional voice, peer support, ethical consultation, and fair organizational processes. Abou Hashish and Razek (2025) reported associations between supportive ethical climates, lower moral distress, and better well-being. However, the present cross-sectional findings do not establish the effectiveness of any particular organizational intervention.

Nursing education and professional development programs could continue to develop conscientious intelligence while also preparing nurses to manage ethical conflict, moral distress, and harmful leadership behavior. Educators could also consider providing training in moral resilience, ethical advocacy, conflict management, communication with authority figures, and appropriate reporting procedures. Aghakhani et al. (2025) emphasized the relevance of both moral sensitivity and resilience to ethical nursing practice under challenging conditions. Individual-focused education should complement, rather than replace, organizational accountability for leadership behavior.

### 5.2 Strengths and limitations of the study

We examined an underexplored model linking toxic leadership, conscientious intelligence, and quality of nursing work life among RNs and used established instruments.

Several limitations should be acknowledged. First, the cross-sectional design prevents conclusions about causality or temporal ordering; therefore, the findings should be interpreted as associations rather than effects. Second, all variables were assessed through self-administered questionnaires completed by the same participants at one time point, creating a risk of common method variance and response bias. Anonymity, sealed envelopes, neutral instructions, and validated instruments were used to limit this risk but could not eliminate it. Third, the sample was drawn from one institutional context in Egypt, which may limit the generalizability of the findings to other organizations, regions, and healthcare systems. Fourth, the small numbers in some age and educational groups require caution when interpreting demographic differences. Fifth, the analyses focused on total construct scores and may have obscured differences among the separate dimensions of toxic leadership, conscientious intelligence, and quality of nursing work life. Finally, the modest interaction may be sample-specific, and statistical moderation in cross-sectional data does not establish a causal moderating process.

Future researchers should use longitudinal, multi-site, and multi-source designs to examine the temporal ordering and robustness of these associations across healthcare settings. Researchers should also examine the separate dimensions of the three constructs and assess contextual factors such as ethical climate, staffing adequacy, organizational justice, and peer support. Future researchers could consider including toxic leadership, conscientious intelligence, ethical climate, and moral distress within the same model to test the proposed explanation for the unexpected interaction pattern.

## 6 Conclusion

Toxic leadership was negatively associated with quality of nursing work life, whereas conscientious intelligence showed a positive bivariate association. Conscientious intelligence significantly moderated the association between toxic leadership and quality of nursing work life but not in the hypothesized protective direction. Instead, the negative association became stronger as conscientious intelligence increased. One possible interpretation is that ethically aware participants were more sensitive to perceived violations of professional and moral values, although this explanation requires direct testing. Efforts to improve the quality of nursing work life should consider including organizational strategies that address toxic leadership and promote supportive ethical climates rather than relying solely on nurses’ individual moral resources.

## Ethics declaration

Informed-consent: Written informed consent to take part in the study and to publish the article has been obtained from all participants or their legal representatives. The privacy rights of participants have been observed.

Human-study: This study was performed in compliance with relevant laws, regulatory frameworks and guidelines where the research took place.

This study was approved by the Research Ethics Committee.

(Approval No KFSIRB400–67)

## CRediT authorship contribution statement

**Ali D. Abousoliman:** Writing – review & editing, Writing – original draft, Visualization, Validation, Supervision, Formal analysis, Data curation, Conceptualization. **Mohamed Gamal Noaman Malek:** Resources, Methodology, Investigation. **Hagar Mahmoud Hamed:** Software, Resources, Project administration, Methodology, Investigation.

## Declaration of competing interest

The authors declare that they have no known competing financial interests or personal relationships that could have appeared to influence the work reported in this paper.

## Data Availability

The data that support the findings of this study are available from the corresponding author upon reasonable request.

## References

[bib0001] Abou Hashish E.A., Razek N.M.F.A. (2025). Navigating connections: how moral resilience shapes ethical climate, distress, and courage among nurses. Sage Open.

[bib0002] Aghakhani N., Ewalds-Kvist B.M., Aghakhani S., Abolfathpour P. (2025). Navigating ethical challenges: the role of moral distress, sensitivity, and resilience in the nursing profession. SAGe Open Nurs..

[bib0003] Ahmed A.K., Atta M.H.R., El-Monshed A.H., Mohamed A.I. (2024). The effect of toxic leadership on workplace deviance: the mediating effect of emotional exhaustion, and the moderating effect of organizational cynicism. BMC Nurs..

[bib0004] Aktı Ü., Giderler C., Duran C., Behdioğlu S., Boz D. (2017). The conscientious intelligence scale: the study of its validity and reliability. İslam Medeniyeti Araştırmaları Dergisi.

[bib0005] Alsadaan N., Alqahtani M. (2024). Toxic leadership in emergency nurses: assessing abusive supervision and its team-level impacts on conflict management and organizational commitment. J. Nurs. Manage.

[bib0006] Arslanoğlu A., Çengel Ö., Gemlİk N., Yilmaz K., Maden Ö (2025). The relationship between conscientious intelligence and human values: application in the field of health. Psychology. Health Medicine.

[bib0007] Bakker A.B., Demerouti E. (2017). Job demands–resources theory: taking stock and looking forward. J. Occup. Health Psychol..

[bib0008] Baron R.M., Kenny D.A. (1986). The moderator–mediator variable distinction in social psychological research: conceptual, strategic, and statistical considerations. J. Pers. Soc. Psychol..

[bib0009] Brooks B.A., Anderson M.A. (2005). Defining quality of nursing work life. Nurs. Econ..

[bib0010] Brooks B.A., Storfjell J., Omoike O., Ohlson S., Stemler I., Shaver J., Brown A. (2007). Assessing the quality of nursing work life. Nurs. Adm. Q..

[bib0011] Conroy N., Patton D., Moore Z., O'Connor T., Nugent L., Derwin R. (2023). The relationship between transformational leadership and staff nurse retention in hospital settings: a systematic review. BioMed. Res. Int..

[bib0012] Dur Ş., Göl İ., Erkin Ö. (2022). The effects of nursing students' conscientious intelligence on their cultural sensitivity levels. Perspect. Psychiatr. Care.

[bib0013] Faraji O., Salehnejad G., Gahramani S., Valiee S. (2017). The relation between nurses' quality of work life with intention to leave their job. Nurs. Practice Today.

[bib0015] Koç O., Şahin H., Öngel G., Günsel A., Schermer J.A. (2022). Examining nurses' vengeful behaviors: the effects of toxic leadership and psychological well-being. Behav. Sci..

[bib0016] Labrague L.J. (2024). Linking toxic leadership with work satisfaction and psychological distress in emergency nurses: the mediating role of work-family conflict. J. Emerg. Nurs..

[bib0018] Lee Y.W., Dai Y.T., Chang M.Y., Chang Y.C., Yao K.G., Liu M.C. (2017). Quality of work life, nurses' intention to leave the profession, and nurses leaving the profession: a one-year prospective survey. J. Nurs. Scholarship.

[bib0019] Mrayyan M.T. (2026). Effects of nursing leaders' toxic leadership on nurses' workplace satisfaction, job engagement, and turnover intention: an online cross-sectional study. J. Adv. Nurs..

[bib0020] Ofei A.M.A., Poku C.A., Paarima Y., Barnes T., Kwashie A.A. (2023). Toxic leadership behaviour of nurse managers and turnover intentions: the mediating role of job satisfaction. BMC Nurs..

[bib0022] Özcan Ş., Yanmış S. (2022). Have we been able to improve the conscientious intelligence levels of nursing students sufficiently?. Perspect. Psychiatr. Care.

[bib0023] Schmidt A.A. (2008).

[bib0024] Tamata A.T., Mohammadnezhad M. (2023). A systematic review study on the factors affecting shortage of nursing workforce in the hospitals. Nurs. Open.

[bib0025] Tawfik A.F., El-Ashry A.M., Atalla A.D.G., Mahgoub S.A.E.F. (2025). Toxic leadership and quality of work life: the moderating role of nurses' agility. BMC Nurs..

[bib0026] World Health Organization (2020). https://www.who.int/publications/i/item/9789240003279.

[bib0027] Xue K., Shang J., Yang C., Pan L., Shi H., Zeng Y. (2025). Nurses' moral distress and ethical climate: a systematic review and meta-analysis. J. Res. Nurs..

[bib0028] Yang Y., Guo X., Zhang M., Liu X., Zhang X. (2023). The impact of resilience on clinical nurses' moral courage during COVID-19: a moderated mediation model of ethical climate and moral distress. Int. Nurs. Rev..

